# Concurrent fulminant type 1 diabetes and typhoid fever: A case report

**DOI:** 10.1097/MD.0000000000044316

**Published:** 2025-09-19

**Authors:** Lihua Fang, Runtian Chen, Qingxian Li, Dan Liu, Jie Ning

**Affiliations:** a Department of Endocrinology, Shenzhen Longhua District Central Hospital, Shenzhen, Guangdong Province, China.

**Keywords:** diabetic ketoacidosis, diarrhea, FT1DM, infectious diseases, typhoid fever

## Abstract

**Rationale:**

Fulminant type 1 diabetes mellitus (FT1DM) is a hyperacute form of diabetes characterized by rapid β-cell destruction. It is often underdiagnosed due to its varied presentations. Typhoid fever, a life-threatening systemic infection caused by *Salmonella enterica serovar Typhi (S. Typhi*), has been infrequently documented in association with FT1DM. This case stress the importance of early diagnosis and aggressive management of FT1DM, particularly when triggered by infectious diseases like typhoid fever.

**Patient concerns:**

A 33-year-old male presented with high fever, persistent pruritic rash, generalized lymphadenopathy, severe diarrhea, gastrointestinal bleeding, and acute hyperglycemic crisis with ketoacidosis.

**Diagnoses:**

Extensive diagnostic workup, including laboratory tests, microbiological cultures, and serological profiling, confirmed the diagnosis of FT1DM complicated by typhoid fever.

**Interventions:**

The patient was treated with an intensive regimen designed to stabilize his metabolic status and manage the infection.

**Outcomes:**

The comprehensive therapeutic intervention was successful, with the patient’s symptoms being alleviated and his condition stabilized.

**Lessons:**

This case underscores the importance of considering FT1DM in acute metabolic emergencies with infectious comorbidity, the necessity for thorough microbiological evaluation in febrile diabetic crises, and the potential pathogenic interplay between typhoid fever and FT1DM onset.

## 1. Introduction

Fulminant type 1 diabetes mellitus (FT1DM), a hyperacute form of diabetes characterized by rapid β-cell destruction, was first clinically delineated in 2000 based on Japanese case series.^[1]^ Distinguished from classical type 1 diabetes by its abrupt presentation and universal absence of diabetes-associated autoantibodies, this entity has shown distinct epidemiological patterns with 89% of reported cases originating from East Asia.^[2]^ Pathophysiological understanding has evolved through multicenter cohort studies, revealing a progression from initial flu-like symptoms to a hyperglycemic crisis, and ultimately to permanent insulin dependence.^[3]^ Emerging evidence implicates multifactorial pathogenesis involving HLA-DR4/DQ4 haplotypes, viral mimicry mechanisms, and immune checkpoint inhibitor-induced PD-1/PD-L1 axis disruption.^[4]^ Notably, the 2023 ENDBASE registry data demonstrate female predominance and bimodal age distribution peaking at 25 to 34 and 55 to 64 years. Critical management requires protocolized monitoring of serum 1,5-anhydroglucitol (<10 μg/mL predicts FT1DM with 92% specificity) coupled with continuous glucose monitoring during suspected β-cell destruction phases.^[5]^

Typhoid fever, a life-threatening systemic infection caused by *Salmonella enterica serovar Typhi (S. Typhi*), has been infrequently documented in association with FT1DM, a condition characterized by abrupt pancreatic β-cell destruction.^[6]^ *Salmonella* infections, particularly those induced by *S. Typhi* and *S. enterica serovar Paratyphi A (S. Paratyphi A*), remain critical public health burdens in endemic regions, with an estimated 11 to 21 million annual cases globally.^[7]^ Epidemiological surveillance reveals distinct geographical serovar distributions: multidrug-resistant *S. Typhi* strains dominate in Asia, whereas drug-resistant *S. Paratyphi A* demonstrates escalating prevalence.^[8]^ Untreated cases progress to severe complications, including intestinal perforation (1–3% incidence), septic shock, and mortality rates exceeding 20% in high-risk populations.^[9]^ In resource-constrained settings lacking blood culture capabilities, typhoid fever diagnosis relies on composite criteria integrating clinical syndromic patterns, serological confirmation via Widal testing (O-antigen titers ≥ 1:160), and systematic exclusion of alternative febrile etiologies.^[10]^ This case study firstly investigates the diagnostic complexities and multidisciplinary management strategies for concurrent FT1DM and typhoid fever comorbidity, emphasizing pathophysiological interplay between systemic bacteremia and autoimmune β-cell dysfunction.

## 2. Case report

### 2.1. Case presentation

A 33-year-old male factory worker with no significant medical history presented with high fever (39 °C), persistent pruritic rash, and generalized lymphadenopathy. He denied toxic substance exposure but reported chronic tobacco use (15 pack-years) and regular alcohol consumption (10 years). Physical examination revealed cachexia (body mass index 18.3 kg/m^2^; height 162 cm, weight 48 kg), tachycardia (110 bpm), and normotension (101/77 mm Hg). Clinical findings included diffuse palpable lymph nodes, hepatosplenomegaly on abdominal ultrasonography, and a widespread rash involving the face, trunk, and extremities. Cardiopulmonary examination was unremarkable without peripheral edema. Treatment with antihistamines and corticosteroids led to gradual resolution of rash and fever within 10 days. Despite comprehensive evaluation, the etiology remained undetermined, culminating in the patient’s self-discharge against medical advice prior to diagnostic confirmation.

Subsequently, the patient experienced clinical deterioration, necessitating a return visit to our emergency department due to diarrhea and gastrointestinal bleeding. Laboratory tests were instrumental in unveiling the metabolic derangements present, with a venous blood glucose level markedly elevated at 41.37 mmol/L, as illustrated in Table 1, consistent with severe hyperglycemia. Biochemical evidence of diabetic ketoacidosis (DKA) was confirmed by a β-hydroxybutyrate level of 6.01 mmol/L and bicarbonate level of 15 mmol/L, findings strongly suggestive of T1DM given the acute disease progression. The clinical presentation was characterized by the triad of severe diarrhea, hyperglycemic crisis, and ketoacidosis. Notably, complete blood count demonstrated a paradoxical significant reduction in both absolute eosinophil count (0.01 × 10^9^/L) and relative proportion (0.01%), accompanied by elevated high-sensitivity C-reactive protein (10.56 mg/L), suggesting concurrent systemic inflammation. The patient was subsequently admitted to the endocrinology unit for intensive management of DKA and comprehensive evaluation of the unexplained gastrointestinal manifestations.

**Table 1:** Laboratory findings at presentation.

Parameter	Result	Reference range
Venous blood gas (ambient air)		
pH	7.33	7.35–7.45
pCO_2_, mm Hg	19.2	35–45
HCO_3_^−^, mmol/L	15	22–26
Base excess, mmol/L	−15.7	−3 to 3
Lactic acid, mmol/L	2.0	0.5–1.6
Biochemistry		
Plasma glucose, mmol/L	41.37	3.9–6.1
Plasma ketones, mmol/L	6.1	0.02–0.27
Serum potassium,mmol/L	6.25	3.5–5.1
Blood sodium, mmol/L	120	137–145
Bicarbonate radical, mmol/L	9.9	22–30
HbA1c, %	5.6	4–6
Fasting insulin, pmol/L	<1.39	17.8–173
Fasting C-peptide, nmol/L	0	0.3–1.47
ICA	Negative	Negative
IA2-Ab	0.79	0–1
GAD-Ab	1.57	0–5
IAA	Negative	Negative
Cortisol, nmol/L (0 am)	322.3	68.2–327 (4:00 pm–8:00 pm)
Leukocytes	14.1*10^9^	3.5–9.5
NEUT, %	67.5	51–75
Eosinophilic	0.01*10^9^	0.05–0.3
EO, %	0.1	0.5–5.0
Hemoglobin, g/L	143*10^9^	>135
Platelets	461*10^9^	>167
Hs-CRP, mg/L	54.1	0–10
PCT, ng/mL	0.22	0–0.5
ESR	20	0–15
Occult blood test	Positive	Negative

This table summarizes the initial laboratory findings of the patient at the time of presentation, including blood gas analysis, biochemistry results, and various antibody tests. The reference ranges are provided for comparison. These findings were crucial in diagnosing diabetic ketoacidosis and ruling out other potential causes of hyperglycemia.

EO = eosinophil percentage, ESR = erythrocyte sedimentation rate, GAD-Ab = glutamic acid decarboxylase antibody, HbA1c = glycated hemoglobin, HCO_3_^−^ = bicarbonate ion, Hs-CRP = high-sensitivity C-reactive protein, IA2-Ab = anti-tyrosine phosphatase antibody, IAA = anti-insulin autoantibody, ICA = anti-islet cell antibody, NEUT = neutrophil percentage, pCO_2_ = partial pressure of carbon dioxide, PCT = procalcitonin, PH = potential of hydrogen.

### 2.2. Diagnostic methods

The patient underwent a comprehensive diagnostic workup to identify the underlying causes of his symptoms. Laboratory investigations included blood gas analysis, biochemistry tests, and various antibody assays. Venous blood gas analysis revealed metabolic acidosis with a pH of 7.33, a partial pressure of carbon dioxide (pCO_2_) of 19.2 mm Hg, and a bicarbonate level (HCO_3_^−^) of 15 mmol/L. Biochemistry results showed severe hyperglycemia with a plasma glucose level of 41.37 mmol/L and elevated ketone bodies at 6.1 mmol/L. Additionally, the patient had a near-normal glycated hemoglobin level of 5.6%, undetectable fasting insulin, and no detectable diabetes-related autoantibodies. These findings met the Imagawa criteria for FT1DM.

Infectious etiologies were extensively evaluated through viral antigen and antibody tests, as well as bacterial cultures from multiple sites, including sputum, urine, stool, bone marrow, and blood (Table 2). All cultures were negative. Serological testing, however, revealed a positive Widal test with anti-O titers of 1:640 and anti-H titers of 1:640, confirming the diagnosis of typhoid fever despite negative confirmatory cultures. This fulfilled the modified case definition for culture-negative enteric fever in endemic areas where a ≥4-fold titer rise cannot be documented. The diagnostic challenge was compounded by overlapping inflammatory markers between FT1DM and systemic salmonellosis, necessitating a careful differentiation between the 2 conditions.

**Table 2:** Etiological testing for infections.

Parameter	Result	Reference range
COVID-19 antigen	Negative	Negative
FluAV-Ig	Negative	Negative
FluBV-Ig	Negative	Negative
EBV-DNA, copies/mL	<500	<500
HSV I IgM	Negative	Negative
HSV II IgM	Negative	Negative
CMV IgM	Negative	Negative
RV IgM	Negative	Negative
TOX IgM	Negative	Negative
Anti-HIV 1 and 2	Negative	Negative
Liver fluke IgG	Negative	Negative
Schistosome IgG	Negative	Negative
Sputum cultures	Negative	Negative
Urine cultures	Negative	Negative
Stool cultures	Negative	Negative
Bone marrow cultures	Negative	Negative
Blood cultures	Negative	Negative

This table details the results of various etiological tests performed to identify the source of infection in the patient. These tests include viral antigen and antibody tests, as well as bacterial cultures from different sites. The negative results helped to narrow down the diagnosis to typhoid fever based on serological testing.

Anti-HIV 1 and 2 = anti-HIV-1 and HIV-2 antibodies, CMV IgM = cytomegalovirus IgM antibody, EBV-DNA = Epstein–Barr virus DNA, FluAV-Ig = influenza A virus antigen, FluBV-Ig = influenza B virus antigen, HSV I IgM = herpes simplex virus type 1 IgM antibody, HSV II IgM = herpes simplex virus type 2 IgM antibody, RV IgM = rubella virus IgM antibody, TOX IgM = toxoplasma gondii IgM antibody.

Follow-up serological testing was performed to monitor the progression of the typhoid infection. The Widal test results showed a dynamic change in antibody titers over time, with initial high titers of anti-O (1:640) and anti-H (1:640) that gradually decreased to anti-O (1:160) and anti-H (1:640) after treatment. This confirmed the diagnosis of typhoid fever and demonstrated the effectiveness of the antimicrobial treatment.

### 2.3. Treatment and follow-up

The patient was treated with a multifaceted therapeutic approach to address both DKA and typhoid fever. Initial management involved intravenous insulin infusion to rapidly correct hyperglycemia and electrolyte imbalances associated with DKA. This was followed by a transition to a subcutaneous basal-bolus insulin regimen, consisting of glargine insulin (10 units nightly) for basal coverage and aspart insulin (6 units preprandially) to manage mealtime glucose excursions. Concurrently, antimicrobial therapy was initiated with intravenous levofloxacin (500 mg daily), based on local susceptibility patterns for *Salmonella Typhi*. This regimen was consistent with current guidelines for the management of typhoid fever.

The patient’s insulin dosages were meticulously adjusted based on continuous glucose monitoring to ensure tight glycemic control while minimizing the risk of hypoglycemia. Follow-up laboratory tests showed a gradual decrease in Widal test titers, as shown in Table 3, indicating a favorable response to antimicrobial therapy. Glycemic control was maintained with fasting blood glucose levels within the target range.

**Table 3:** Follow-up Widal test results.

Date	Parameter	Result	Reference range
6–10	Wida-O	Negative	Negative < 1:80
	Wida-H	Negative	Negative < 1:160
	Wida-A	Negative	Negative < 1:80
	Wida-B	Negative	Negative < 1:80
	Wida-C	Negative	Negative < 1:80
6–22	Wida-O	Positive (1:640)	Negative < 1:80
	Wida-H	Positive (1:640)	Negative < 1:160
	Wida-A	Negative	Negative < 1:80
	Wida-B	Negative	Negative < 1:80
	Wida-C	Positive (1:320)	Negative < 1:80
7–06	Wida-O	Positive (1:160)	Negative < 1:80
	Wida-H	Positive (1:640)	Negative < 1:160
	Wida-A	Negative	Negative < 1:80
	Wida-B	Negative	Negative < 1:80
	Wida-C	Positive (1:160)	Negative < 1:80

This table presents the Widal test results over time, showing the dynamic changes in antibody titers against *Salmonella Typhi*. The progressive increase and subsequent decline in titers confirmed the diagnosis of typhoid fever and demonstrated the effectiveness of the antimicrobial treatment.

Upon discharge, the patient continued on the basal-bolus insulin regimen with a plan for close follow-up. Follow-up assessments conducted 3 weeks post-discharge revealed persistently low C-peptide levels, confirming the diagnosis of FT1DM and the need for ongoing exogenous insulin therapy. The patient demonstrated good adherence to the prescribed regimen and reported no adverse events related to insulin or antimicrobial therapy. During follow-up, the patient expressed satisfaction with the prognosis and the management of his condition.

## 3. Discussion

The pathogenesis of FT1DM is not fully understood but is thought to involve genetic predisposition,^[11]^ viral infections,^[12]^ and possibly an autoimmune component^[13]^ or drug effector^[14]^ and here may related with bacterial infections like typhoid fever. The rapid onset and severity of FT1DM make it a medical emergency that requires prompt recognition and treatment to prevent severe complications and death. The inflammatory cascade induced by *S. Typhi* infection provides a plausible pathway for β-cell destruction. Elevated IL-6 and TNF-α levels characteristic of typhoid fever correlate with experimental models showing cytokine-mediated β-cell apoptosis via oxidative stress pathways.^[15]^ These proinflammatory mediators also induce insulin resistance through JNK-1 activation in peripheral tissues, creating a dual metabolic insult that may overwhelm marginal β-cell reserves in predisposed individuals.^[16]^ The mechanism aligns with reports of stress-induced hyperglycemia progressing to irreversible diabetes in sepsis models, though typhoid-specific evidence remains scarce.^[17]^

Metabolic stressors unique to typhoid infection further compound this risk. The characteristic hypermetabolic state, driven by surges in cortisol and catecholamines, may lead to β-cell exhaustion through persistent glucolipotoxicity.^[18]^ In individuals with HLA-DR4 haplotype or TCF7L2 polymorphisms,^[19]^ this metabolic overload could theoretically unmask latent autoimmune diabetes through epitope spreading mechanisms.^[20]^ However, the absence of documented FT1DM cases directly attributable to typhoid infection underscores the need for population-based studies to validate these pathophysiological models.^[21]^ Clinically, these observations warrant heightened surveillance for glucose dysregulation in typhoid-endemic regions.^[22]^ The delayed diagnosis of FT1DM in resource-limited settings emphasizes the need for protocolized glycated hemoglobin and C-peptide monitoring during acute infections.^[23]^

Preventive strategies targeting cytokine modulation, such as IL-1β antagonists, merit investigation given their proven efficacy in post-pancreatitis diabetes prevention.^[24,25]^ While current evidence remains circumstantial, several key research priorities have emerged. These include conducting prospective studies to correlate the severity of typhoid fever with declines in β-cell function, performing immunohistochemical analyses on pancreatic tissue from fatal typhoid cases, and conducting genome-wide association studies to identify susceptibility loci for infection-associated FT1DM. Until such data become available, clinicians should maintain a high suspicion for autoimmune diabetes in patients experiencing prolonged hyperglycemia following typhoid fever. Future studies should focus on longitudinal assessments of β-cell function in typhoid survivors and evaluate cytokine-blocking therapies as preventive strategies. Clinicians managing prolonged post-typhoid hyperglycemia should consider FT1DM screening while acknowledging the current evidence remains preliminary. Bridging infectious disease and diabetology frameworks may advance therapeutic interventions for infection-related metabolic crises.

## 4. Conclusion

**Figure 1.**
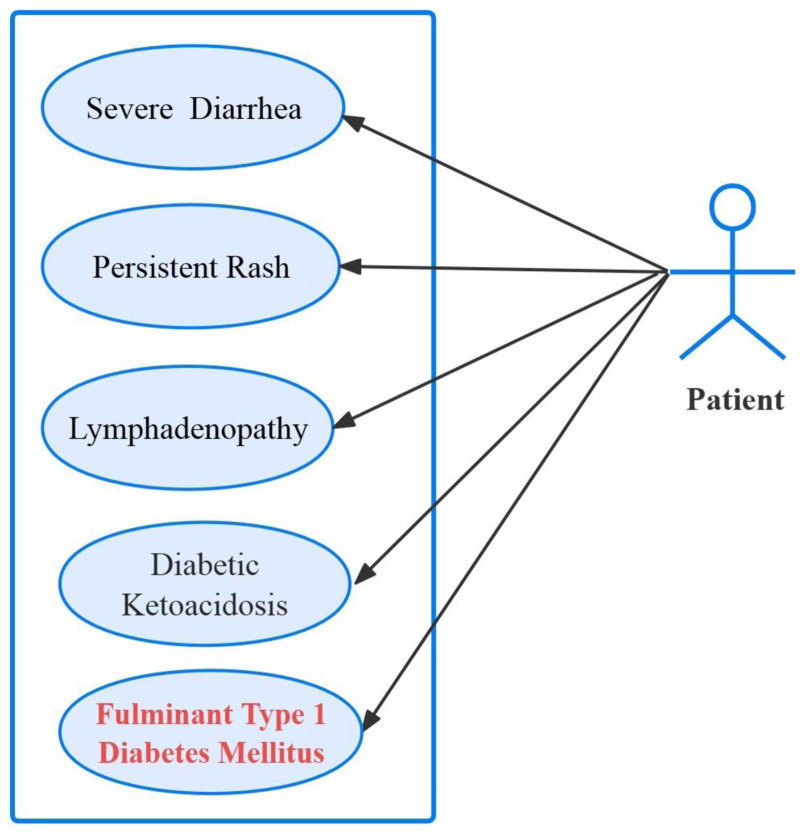
Fluminant type 1 diabetes mellitus induced by typhoid fever.

This case of concurrent FT1DM and typhoid fever underscores the intricate relationship between infection and autoimmune processes in the pathogenesis of FT1DM as shown in Figure 1. Early recognition and aggressive management, including prompt initiation of insulin therapy and targeted antimicrobial treatment, are crucial for stabilizing the acute metabolic crisis and preventing long-term complications. This report emphasizes the importance of maintaining a high index of suspicion for FT1DM in patients with prolonged hyperglycemia following typhoid fever, particularly in endemic regions. Forthcoming research should focus on elucidating the mechanisms underlying the interplay between infections and autoimmune diabetes to inform more targeted therapeutic strategies.

## Acknowledgments

The authors appreciate the patient’s consent to present this case.

## Author contributions

**Conceptualization:** Runtian Chen, Qingxian Li.

**Investigation:** Runtian Chen, Qingxian Li, Dan Liu.

**Methodology:** Qingxian Li, Dan Liu.

**Supervision:** Jie Ning.

**Validation:** Lihua Fang, Dan Liu.

**Writing – original draft:** Lihua Fang.

**Writing – review & editing:** Lihua Fang, Runtian Chen.
